# Use of Human Serum Albumin Cys^34^ (HSA-Cys^34^) Adductomics as a Multidimensional and Integrative Biomarker Approach to Assess Oxidative Stress

**DOI:** 10.3390/antiox15040458

**Published:** 2026-04-08

**Authors:** Aishwarya Jala, Fariba Tayyari, William E. Funk

**Affiliations:** Department of Preventive Medicine, Feinberg School of Medicine, Northwestern University, Chicago, IL 60611, USA; aishwarya.jala@northwestern.edu (A.J.); ftayyari@northwestern.edu (F.T.)

**Keywords:** human serum albumin (HSA), Cys^34^, oxidative stress, adductomics, biomarkers, reactive oxygen species (ROS), redox biology

## Abstract

Human serum albumin (HSA) is the most abundant protein in plasma, and the redox state of circulating HSA has been used as a biomarker of systemic oxidative stress (OS) for decades. While informative, many traditional biomarkers of OS measure short-lived or downstream products of oxidative damage that offer limited perspectives on the dynamic and integrated processes that govern systemic redox biology within human populations. By moving beyond single-analyte damage markers and towards coordinated patterns of protein modifications, HSA-Cys^34^ adductomics offers a systems-level approach that simultaneously captures change in multiple layers of OS. Because of its high abundance in plasma and HSA’s unique and highly reactive single free thiol (Cys^34^), HSA-Cys^34^ serves as an ideal sentinel target for monitoring reactions with reactive oxygen species (ROS), reactive nitrogen species (RNS), and electrophilic species produced by endogenous metabolism and responses to exogenous chemical exposures. The reaction of HSA with ROS, RNS, and reactive electrophiles yields a diverse array of protein modifications, including direct oxidation products (sulfenic, sulfinic, and sulfonic acid), low molecular weight thiol-disulfide exchange, and lipid peroxidation (LPO)-derived reactive aldehydes. With a mean residence time of about a month, these accumulated adducts serve as an integrated picture of oxidative and electrophilic stress that together function as a molecular record of systemic redox physiology. Previous studies using high-resolution mass spectrometry-based adductomics have enabled global untargeted analysis of HSA-Cys^34^ modifications, yielding an expansive inventory of novel redox signatures of environmental stressors and disease states. In this paper we review the chemistry and biology underlying OS-related modifications of HSA-Cys^34^ and highlight the important role of HSA-Cys^34^ adducts as integrative biomarkers of OS at the interface of molecular biology, exposure assessment, and public health research.

## 1. Introduction

Redox homeostasis (oxidation-reduction), like pH regulation, is central to many biological processes. Oxidative stress (OS) is defined as “an imbalance between oxidants and antioxidants in favor of the oxidants, leading to a disruption of redox signaling and control and/or molecular damage” [1,2].

Physiological (low-level) OS plays a critical role in redox regulation (oxidative eustress), whereas supraphysiological oxidative challenge can result in damage to macromolecules and disrupt redox signaling (oxidative distress) [3]. OS is an important component of various diseases, including cancer, cardiovascular disorders, diabetes, and neurodegeneration [4,5,6,7,8,9]. Numerous methods have been developed and applied to measure the extent and nature of OS across multiple biological layers, including direct molecular oxidation, antioxidant capacity, lipid peroxidation, and redox homeodynamics [10]. Direct molecular oxidation is commonly assessed through measurements of oxidized DNA (e.g., 8-oxo-2′-deoxyguanosine), protein carbonyls, and lipid oxidation products. Antioxidant capacity is evaluated using assays such as total antioxidant capacity (TAC), glutathione levels, and superoxide dismutase (SOD) activity. Lipid peroxidation is measured through biomarkers like malondialdehyde (MDA), 4-hydroxynonenal (4-HNE), and isoprostanes. Redox homeodynamics can be assessed through dynamic measurements of redox couples (e.g., GSH/GSSG ratio) and redox potential. Although a plethora of OS biomarkers have been used in research studies, clinical applications have been limited due to non-specificity and inconsistent findings across biomarker applications [10,11,12,13]. Critically, these conventional OS biomarkers each capture only a single layer of OS. For example, malondialdehyde reflects lipid peroxidation, 8-oxo-2′-deoxyguanosine reflects direct DNA oxidation, and protein carbonyls reflect direct protein oxidation and do not provide an integrated assessment across all four layers of OS biology, a gap that human serum albumin Cys^34^ (HSA-Cys^34^) adducts are uniquely positioned to address.

Site-specific modifications of proteins represent a key molecular mechanism for transforming an oxidant signal into a biological response [14]. Protein cysteine thiols serve as major sensors for elevated ROS levels. Redox reactions with protein cysteine thiols result in a range of post-translational modifications. Depending on the levels of ROS and the local protein cysteine environment, protein thiols can be covalently modified reversibly or irreversibly. While the reversible modifications, such as sulfenic acids, disulfides, glutathionylation, thiosulfinates, and sulfenamides, modulate protein function and act as redox switches in cellular signaling [14]. Irreversible protein modifications, such as sulfinic acids, sulfonic acids, and sulfonamides, can lead to protein denaturation and serve as markers of protein damage. These modifications are considered the hallmarks of OS and disease.

Among the 214,000 cysteine residues in the human genome, a relatively small subset is involved in redox-related biological processes, often acting as functional ‘switches’ [15]. Within this framework, the only free thiol of human serum albumin, Cys^34^, stands out as a highly reactive and abundant residue that reflects systemic oxidative and electrophilic stress. Beyond its antioxidant capacity, HSA serves multiple physiological functions, including maintenance of oncotic pressure, transport of fatty acids, metals, and drugs, pseudo-enzymatic activities, and acid-base balance [16]. While the specific role of Cys^34^ as a functional redox switch remains to be established, the stability and abundance of HSA-Cys^34^ modifications (adducts) provide a novel biomarker approach for gaining insights into redox status and investigating disease-related oxidative imbalances.

This scoping review maps current knowledge on HSA-Cys^34^ adductomics, including mechanisms of chemical formation, biological significance, and current literature on HSA-Cys^34^ adducts, highlighting their important role as integrative biomarkers of oxidative and electrophilic stress.

## 2. HSA-Cys^34^ Adductomics

**Figure 1:**
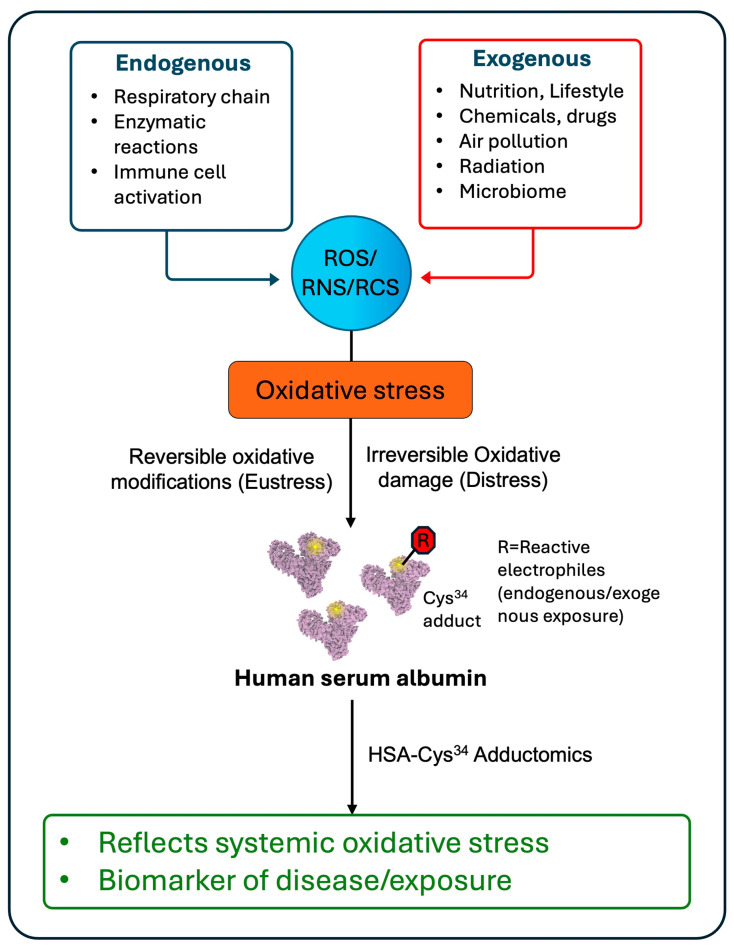
Schematic representation of oxidative stress and HSA-Cys^34^ adductomics. Reactive oxygen, nitrogen, and carbonyl species (ROS/RNS/RCS) arise from both endogenous and exogenous sources, leading to oxidative stress and formation of adducts on the highly nucleophilic Cys^34^ residue of human serum albumin (HSA). HSA-Cys34 adductomics captures systemic oxidative distress and eustress and serves as a novel biomarker for investigating links between environmental exposures and adverse health outcomes.

Protein adductomics is the analysis of the totality of adducts bound to blood proteins. HSA-Cys^34^, the only free sulfhydryl group in HSA, is an exceptional nucleophilic hotspot, which offers distinct advantages for adductomics research (i.e., HSA-Cys^34^ adductomics) [17]. This approach serves as an unbiased method for quantifying a vast range of adducts with circulating electrophiles and other reactive small molecules from both exogenous and endogenous sources (Figure 1).

HSA is a predominant protein in blood, with a mean residence time of 28 days, making up nearly 60% (35–50 g/L) of all proteins present in circulating plasma. HSA consists of 35 cysteine residues, 34 of which are involved in the formation of disulfide bonds. Cys^34^ is the only free cysteine residue in HSA and represents ~80% of all free thiols in human plasma. HSA-Cys^34^ is critical for the antioxidant activity of HSA, serving as the primary plasma scavenger of reactive chemical species [18,19,20]. In healthy individuals, about 70–80% of HSA-Cys^34^ exists in the free thiol form (mercaptalbumin) [17], 25% of HSA-Cys^34^ as disulfides with low molecular weight (LMW) thiols like cysteine, homocysteine, or glutathione, and a smaller fraction is oxidized to higher oxidation forms such as sulfinic, sulfenic, and sulfonic acid (non-mercaptalbumin) [21,22].

The high reactivity of Cys^34^ is governed by its ionization state and local microenvironment. Under physiological conditions, the pKa of HSA-Cys^34^ is approximately 8.1, which is significantly lower than the typical pKa of peptide thiols (~9.1) [23,24,25]. The lower pKa is explained by the local environment of Cys^34^, comprising Tyr^83^, Glu^33^, and Asp^38^, which forms a stabilizing hydrogen-bonding network [26]. This allows a significant fraction (approximately 17%) of the thiol to exist in its reactive thiolate (HSA-S^−^) form at physiological pH. This unique property is essential for HSA-Cys34’s high nucleophilic activity and enables Cys^34^ to efficiently scavenge reactive oxygen and nitrogen species, electrophiles, and small thiols. Because of this important biological role, this single free thiol on HSA is preserved in all mammalian species [26].

The amino acid residues adjacent to HSA-Cys^34^ are also similar to those of the active site of glutathione *S*-transferase (GST), and the proximity of tyrosine and histidine residues has been shown to increase thiol acidity by accepting protons and stabilizing the thiolate anion [26]. Neighboring residues with positive charge (Lys^41^ in HSA or Arg in GST) can also increase the polarity of the phenol group and stabilize the thiolate anion [26]. This implies that HSA may have auto-enzymatic activity to catalyze the ionization of Cys^34^ and keep it in its reactive thiolate form [26].

Another key aspect regulating the reactivity of HSA-Cys^34^ is that the sulfur atom is moderately exposed in HSA, with Cys^34^ located within a shallow crevice in domain I of HSA. This limits the intermolecular interactions and explains why the formation of covalent dimers is rarely observed. Notably, the thiolate sulfur (HSA-S^−^) has an increased solvent exposure compared to the protonated form (HSA-SH), enhancing its chemical reactivity [24].

## 3. Chemistry of HSA-Cys^34^ Modifications

**Figure 2:**
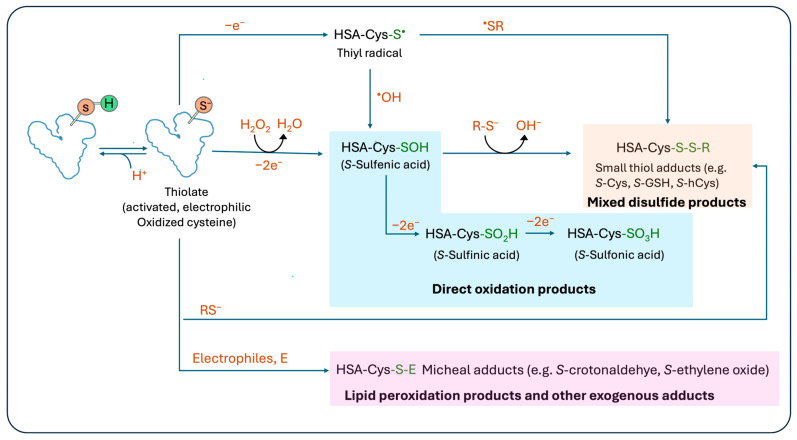
Schematic overview of major oxidative and electrophilic modifications in the HSA-Cys^34^ adductome. The free thiol group of Cys^34^ undergoes sequential oxidation, forming sulfenic (HSA-Cys-SOH), sulfinic (HSA-Cys-SO_2_H), and sulfonic (HSA-Cys-SO_3_H) acids (blue box, direct oxidation products). The sulfenic intermediate and/or thiolate can also engage in thiol-disulfide exchange reactions with low molecular weight thiols (e.g., cysteine, glutathione, homocysteine), generating mixed disulfides (orange box, *S*-Cys, *S*-GSH, *S*-hCys). Alternatively, electrophilic species (E), including reactive lipid peroxidation products and other electrophiles, can form covalent Michael adducts at Cys^34^ (pink box, *S*-crotonaldehyde, *S*-acrolein). These classes of adducts capture a vast range of redox biology that acts together as multidimensional and comprehensive biomarkers of systemic oxidative stress.

Under OS conditions, HSA-Cys^34^ can undergo a range of modifications that capture an integration of transient oxidative and electrophilic stress. Due to its unique properties, Cys^34^ is susceptible to a range of modifications by ROS, RCS (lipid peroxidation products), small thiols, and other reactive electrophiles from exogenous sources. These modifications result in a chemically diverse array of HSA-Cys^34^ adducts that simultaneously capture multiple layers and dimensions of OS (Figure 2).

### 3.1. Formation of HSA-Cys^34^-Sulfenic Acid (S-Sulfenic Acid)

The free thiol (-SH) of HSA-Cys^34^ primarily undergoes two-electron oxidation by oxidants such as hydrogen peroxide or peroxynitrite to form *S*-sulfenic acid. Alternatively, *S*-sulfenic acid is formed from the one-electron oxidation of HSA-Cys^34^ to a thiyl radical, which subsequently reacts with oxygen to produce various secondary radicals, such as peroxyl (R-SOO·) and sulfinyl (R-SO·) radicals, which ultimately lead to sulfenic acid formation [27,28]. Another mechanism involves the hydrolysis of disulfides under alkaline conditions, which can generate sulfenic acid [29,30].

*S*-sulfenic acid is the primary and transient reversible oxidative modification of the thiol group of HSA-Cys^34^. *S*-sulfenic acid exhibits both nucleophilic and electrophilic behavior. Depending on the redox environment, it can react with another thiol to form a disulfide, condense to thiosulfinates, or undergo irreversible oxidation to its sulfinic (-SO_2_H) and sulfonic (-SO_3_H) acid forms. Hence, Cys^34^ serves as an intermediate in the various oxidation pathways. In some cases, *S*-sulfenic acid can also react intramolecularly with the nearby amino acid glutamine (Gln^33^) to form a transient sulfenamide adduct [31], contributing to redox-dependent conformational changes.

### 3.2. Formation of HSA-Sulfinic Acid (S-Sulfinic Acid) and Sulfonic Acid (S-Sulfonic Acid)

Under conditions of prolonged or more severe OS, HSA-SOH can be further oxidized to sulfinic acid (-SO_2_H), which can be oxidized to sulfonic acid (-SO_3_H) [25,32]. The formation of *S*-sulfinic acid and *S*-sulfonic acid was experimentally shown to be influenced by pH, with oxidation to sulfinic and sulfonic acids being favored at alkaline pH due to the ionization of Cys-SOH to Cys-SO^−^ [25]. These higher-oxidation products are stable derivatives and are biologically irreversible. The formation of sulfinic and sulfonic acids is thus indicative of oxidative damage rather than regulatory processes [32].

### 3.3. Formation of HSA-Cys^34^ Small Thiol Adducts (HSA-S-SR)

Cys^34^ disulfides can be formed following one-electron oxidation of the thiol (-SH) group through radical coupling of two thiyl radicals or through reactions involving oxidized intermediates such as sulfenic acid and *S*-nitrosothiols, which can undergo nucleophilic attack by another thiol to yield a disulfide [33]. The thiol (-SH) or sulfenic acid (-SOH) forms of Cys^34^ readily participate in thiol-disulfide exchange reactions with LMW thiols such as cysteine, homocysteine, glutathione, cysteinylglycine, and γ-glutamylcysteine, forming mixed disulfides (HSA-S-SR) [34]. These reactions are reversible and constitute a major mechanism for plasma redox buffering, maintaining dynamic equilibrium between the reduced (HSA-SH) and oxidized (HSA-S-SR) forms.

Mixed disulfide formation is influenced by both the redox potential of the plasma environment and the accessibility of the Cys^34^ pocket. Under physiological conditions, approximately 70–80% of circulating HSA exists in the reduced form (i.e., mercaptalbumin), with the largest oxidized fraction of HSA existing as mixed disulfides. During OS and disease progression, this balance shifts toward the oxidized state, and the ratios of reduced and oxidized HSA-Cys^34^ have long been used as a sensitive biomarker of systemic oxidative burden [21,35,36,37,38].

### 3.4. Cys^34^ Modification by Reactive Aldehydes and Other Chemical Electrophiles

In addition to direct oxidation, HSA-Cys^34^ can form covalent adducts through nucleophilic addition to electrophilic species generated endogenously or from exogenous sources. Lipid peroxidation is a major source of reactive aldehydes, which produce α, β-unsaturated aldehydes, including acrolein, 4-HNE, and crotonaldehyde. These aldehydes act as Michael acceptors and react with the thiolate form of Cys^34^ to form stable thioether adducts.

In addition to lipid-derived aldehydes, reactive drug metabolites, quinones, and environmental toxicants can modify Cys^34^ through conjugate addition or substitution mechanisms. These modifications are generally irreversible and accumulate over the lifespan of HSA, serving as a long-term biomarker of electrophilic and inflammatory stress.

Together, direct oxidation, disulfide exchange, and electrophilic adduction constitute the principal biochemical pathways that comprise the HSA-Cys^34^ adductome. These complex chemical reactions capture a vast array of OS biology, including direct molecular oxidation, antioxidant capacity, lipid peroxidation, and redox homeodynamics that together can offer new insights into the systemic effects of OS. Table 1 summarizes the major classes of OS-related HSA-Cys^34^ adducts, with applications to population-based studies of tobacco smoke, air pollution, occupational exposure, and chronic diseases, and highlights overall trends in relative abundance and directional changes across studies.

## 4. Oxidative Stress Biology Related to HSA-Cys^34^ Modifications

### 4.1. Direct Oxidation Products

The formation of HSA-Cys^34^ sulfenic acid (*S*-sulfenic acid) is a transient intermediate in the subsequent oxidation to HSA-Cys^34^ sulfinic (*S*-sulfinic acid, SO_2_H) and sulfonic (*S*-sulfonic acid, SO_3_H) acids [23,28]. Historically, these oxidation products were presumed to form in vivo but were not detected in human samples, largely due to the high reactivity of sulfenic acid and analytical limitations in resolving low-abundance HSA modifications. Grigoriyan et al. [31] reported direct oxidation products of HSA-Cys^34^ in human plasma and serum, following accurate measurements of T3 peptide, the third largest tryptic peptide of HSA (ALVLIAFAQYLQQC^34^PFEDHVK, 2432 Da), and detecting its oxidized forms as *S*-sulfinic acid (*m*/*z* = 822.4226, +3) and *S*-sulfonic acid (*m*/*z* = 827.7543, +3). Because *S*-sulfenic acid is highly unstable, it is rarely observed directly; instead, sulfinamide adduct (*m*/*z* = 816.4191, +3), formed through the intramolecular reaction of sulfenic acid with adjacent amino acid Gln33, is significantly more stable, which serves as a proxy for *S*-sulfenic acid formation. In a study of smokers and non-smokers, direct oxidation products were readily detected, with the relative abundance of these products in the order sulfinic acid > (possible) sulfinamide > sulfonic acid [31].

While protein direct oxidation products are generally assumed to increase under conditions of elevated OS, recent studies examining the direct oxidation of HSA-Cys^34^ associated with environmental exposures and chronic diseases have consistently reported inverse relationships, highlighting the complexity of the underlying OS biology involved [40,41,42]. This paradox suggests that direct HSA-Cys^34^ oxidation likely involves multiple and competing biochemical processes, including changes in thiol availability, redox buffering capacity, and adaptive responses to sustained oxidative challenge. For instance, workers exposed to benzene, a compound known to induce the production of ROS, resulted in significantly higher levels of all three HSA-Cys^34^ direct oxidation products (*m*/*z* 816.42, 822.42, and 827.76) compared with controls [39], which is consistent with expectations that increased exposures to benzene would result in higher levels of OS-related adducts. However, in contrast, a study of smokers revealed that the levels of *S*-sulfinic acid (and its sodiated form) were significantly lower than levels in non-smokers [41]. It was speculated that this contradictory finding may be due to smoking-induced hypoxia, or alterations in the redox proteome may have suppressed the formation of HSA-Cys^34^ direct oxidation products [41].

These inconsistent findings may be explained in part by dose, where benzene-exposed workers had significantly higher benzene exposures than non-occupationally exposed smokers. The median urinary levels of benzene in nonsmokers and smokers in these studies were 1.36 nM and 2.47 nM, as compared to exposed and unexposed workers with benzene levels of 267 nM and 197 nM, respectively [39]. Given that benzene levels absorbed by cigarette smoking were significantly lower than occupational benzene exposure, the smaller dose of benzene from smoking may not have been sufficient to overcome the counter effects of smoking-induced hypoxia, thus leading to the observed reduced formation of HSA-Cys^34^ direct oxidation products in smokers [41].

A decrease in HSA-Cys^34^ direct oxidation products was also observed in several studies of chronic disease. In one study, HSA-Cys^34^-Gln cross-link (monooxidation), *S*-sulfinic acid, and *S*-sulfinic acid plus methylation were significantly lower in subjects with chronic obstructive pulmonary disease (COPD) and ischemic heart disease (IHD) than in healthy participants [40]. Additionally, *S*-sulfonic acid levels were significantly lower in patients with IHD. A similar decrease in *S*-sulfinic acid levels was reported in males with non-Hodgkin lymphoma compared to controls. Since OS is a hallmark of these diseases, it is surprising that the production of HSA-Cys^34^ direct oxidation products would be decreased in these patients. However, these results consistently align with the proposed hypothesis that hypoxia, which is characteristic of COPD, IHD, and chronic cigarette smoke exposure, causes dysregulation of pathways related to redox control of Cys^34^. Other mechanisms may also be responsible for the consistently lower levels of HSA-Cys^34^ direct oxidation products observed across diseases, particularly in cancer. For example, the sustained activation of antioxidant pathways, including NRF2 signaling and glutathione-dependent buffering, can limit the accumulation of oxidants capable of causing irreversible HSA-Cys^34^ oxidation [51,52,53,54]. Additionally, metabolic reprogramming associated with cancer toward aerobic glycolysis reduces ROS generation in mitochondria, while increased tumor uptake and degradation of HSA may further decrease the circulating pool available for oxidation [55,56,57]. Collectively, hypoxia-related redox dysregulation and cancer-specific adaptive antioxidant and metabolic mechanisms may be responsible for the decreased levels of HSA-Cys^34^ direct oxidation products in chronic diseases and cancer.

Negative relationships have also been observed with direct oxidation products and air pollution exposure, where increased levels of NO_2_ and O_3_ in Central London were associated with decreased Cys^34^ sulfonic acid levels in adult participants [40]. In a larger study investigating air pollution exposure during pregnancy, we reported a similar trend with HSA-Cys^34^ adducts measured in newborn dried blood spots (DBSs). In this study, 120 newborn DBS were obtained from infants born in Southern California, and exposures to PM_2.5_, NO_2_, SO_2_, and O_3_ were estimated using air pollution models. Overall, relationships between air pollution exposures during pregnancy and OS-related HSA-Cys^34^ adducts demonstrated two distinct relationships. First, exposure to O_3_ (a potent oxidant known to generate ROS) during the last 30 days of pregnancy was associated with decreased *S*-sulfinic acid levels [42], consistent with the inverse relationships observed in adults’ exposure to air pollution in Central London [40]. This result was expected since HSA-Cys^34^ adducts in newborn DBS samples can be directly formed with ROS during the last 30 days of pregnancy (i.e., the mean residence time of HSA). Second, air pollution exposure earlier during pregnancy (i.e., 1st and 2nd trimesters) showed the opposite trend. Specifically, levels of *S*-sulfinic acid were increased with maternal O_3_ exposures during the 1st trimester and maternal PM_10_ exposures in the 2nd trimester. These findings suggest that the developing fetus may develop adaptive antioxidant responses when primed with OS in earlier stages of pregnancy, and at the time of birth, these mechanisms may provide protective resilience against perinatal oxidative stressors. This interpretation is consistent with high-altitude pregnancies, where prenatal exposure to hypoxia-induced OS during pregnancy is speculated to prime antioxidant defenses to protect against oxidative damage at the time of birth [58].

In another recent study using cord blood samples, infants with bronchopulmonary dysplasia-associated pulmonary hypertension (BPD-PH) had significantly lower levels of dehydrated *S*-sulfinic acid plus methylation adduct (+CH_3_, -H−O) compared to infants with BPD without PH [44,59]. These findings indicate that BPD-PH may represent a more severe form of OS-mediated lung injury. Unlike BPD-PH, infants exposed to maternal pre-eclampsia (a known risk factor for BPD-PH) had higher levels of *S*-sulfinic acid (+CH_3_, -H−O). In this study, the complex pattern of *S*-sulfinic acid (+CH_3_, -H_2_O) is further complicated by its relationship with the use of postnatal cumulative supplemental oxygen (CSO). At 36 weeks postpartum age, *S*-sulfinic (+CH_3_, -H−O) was lower in infants with high CSO exposure, similar to observed trends with BPD-PH.

Researchers have also applied more holistic analytic approaches to investigate global trends in the HSA-Cys^34^ adductome. Grigoryan et al. employed correlation network analysis of HSA-Cys^34^ adducts from benzene-exposed workers and identified three groups of co-varying HSA-Cys^34^ adducts, including benzene-specific metabolites, ROS/RCS-related modification, and Cys^34^ disulfides [39]. Notably, direct oxidation products (*S*-sulfinic acid, *S*-sulfonic acid, and sulfinamide) grouped together and strongly correlated with reactive aldehyde adducts and protein truncations. Similar patterns have been reported by others. Liu et al. applied multivariate linear regression to assess the joint effects of disease state (COPD, IHD) and residential air pollution exposure on HSA-Cys^34^ adductome and found that multiple oxidation products were reduced in COPD and IHD patients compared to healthy controls [40]. Lin et al. also applied cluster analysis and used heat mapping to visually illustrate that OS-related HSA-Cys^34^ adduct levels from infants with BPD change in a coordinated manner across multiple time points (1 week, 1 month, and 36 weeks postmenstrual age), with 51 adducts significantly increased with high CSO exposure at 1 month and then decreased together by 36 weeks [44]. Patterns of OS-related HSA-Cys^34^ adducts from independent studies demonstrate that changes in the adductome act in concert, which can be used to investigate system-wide changes in OS. Similar to advancements in the field of metabolomics, tracking global changes across classes of adducts may provide valuable insights into underlying dynamic and complex biological processes that would otherwise be missed using single-biomarker approaches. These previous studies emphasize the complex and context-dependent behavior of the HSA-Cys^34^ adductome, which is driven by both systemic OS and protein redox dynamics.

### 4.2. Small Thiols

Beyond their role in antioxidant defense, small thiols play multiple roles in redox regulation [60]. Small thiols directly neutralize ROS and are involved in reversible thiol-disulfide exchange reactions, and their depletion is often used as a marker of increased OS. However, metabolism of thiols is a highly dynamic process, and OS can also induce compensatory upregulation of thiol synthesis, recycling, and turnover of thiols, resulting in elevated levels of specific thiols under certain exposure or disease conditions [61,62].

Contemporary redox biology has redefined OS as a disruption of regulated redox signaling, as opposed to a simple imbalance between oxidants and antioxidants, and within this framework, protein cysteine residues function as key redox switches [61]. Accordingly, changes in levels of HSA-Cys^34^ small thiol adducts can act as sensitive measures of systemic thiol availability, redox status, and protein cysteine regulation, in addition to direct indicators of oxidative burden [31,61]. As a result, the HSA-Cys^34^ adductome can be used to monitor both thiol depletion (oxidative burden) and compensatory responses (adaptation), providing insight into alterations in redox regulatory networks driven by environmental exposures and disease processes [31,40,41,42,43,46,47].

Several HSA-Cys^34^ adductomics studies have demonstrated that small thiol adducts at HSA-Cys^34^ function as coordinated groups that simultaneously capture a vast array of complex OS biology [39,45,47]. Cluster analysis can uncover underlying structures of Cys^34^ disulfides formed with small thiols, including glutathione, γ-glutamylcysteine, cysteine, and related thiols, consistent with shared redox chemistry and interlinked metabolic pathways [39,47]. Similarly, multivariate feature-selection approaches have been applied to investigate diesel engine exhaust exposure, which identified alterations in multiple antioxidant-related thiol adducts, including *S*-GSH and *S*-CysGly, indicating coordinated changes in redox and detoxification processes [45]. Together, these unsupervised approaches can be applied to uncover natural structures within datasets to help understand underlying OS mechanisms linking environmental stressors and adverse health outcomes.

#### 4.2.1. HSA-Cys^34^ Cysteine (*S*-Cys)

Among the LMW thiols, *S*-Cys adducts are the most abundant (∼2.5 nmol adduct per mg HSA). Under physiological conditions, approximately 20–30% of circulating HSA exists as mixed disulfides, with *S*-Cys adducts representing the predominant LMW thiol contributing to this pool [34]. Kinetic studies demonstrate that cysteine reacts rapidly with the sulfenic acid intermediate (HSA-SOH), with a rate constant of 21.6 M^−1^ s^−1^ (pH 7.4, 25 °C), which contributes to the high abundance of *S*-Cys observed in plasma [34]. Cysteine is a proteinogenic amino acid whose circulating levels are governed by protein turnover, dietary intake, sulfur amino acid metabolism, and reduction in cystine, all of which contribute to thiol-disulfide exchange reactions with HSA-Cys^34^. Cysteine in plasma primarily originates from protein catabolism and the transsulfuration pathway, where methionine is converted to cysteine via homocysteine and cystathionine intermediates [63].

Functionally, *S*-Cys serves as a protective, reversible thiol modification that limits further oxidation of HSA-Cys^34^ to irreversible sulfinic or sulfonic acids and reflects changes in circulating thiol redox status. As a result, levels of *S*-Cys adducts can act as sensitive indicators of systemic OS [37]. Variations in the extent of cysteinylation have been associated with several exposure- and disease-related conditions. Adductomics findings have reported decreased levels of *S*-Cys, *S*-Cys+N (a, )and *S*-Cys+K ( i)n smokers [41]. Similarly, exposure to diesel engine exhaust has been associated with significant decreases in the cysteine adduct derivative *S*-Cys (NH_2_→OH) (*m*/*z* = 851.76), indicating perturbations in the systemic thiol redox balance [45].

In the Shanghai Women’s Health Study, *S*-Cys adducts were significantly lower in lung cancer cases than in controls, indicative of depletion of circulating thiol-based buffering capacity and altered redox homeostasis in a population of never-smokers. Similar patterns were also observed with chronic exposure to PM_2.5_ and polycyclic aromatic hydrocarbons (PAH) from ambient air pollution and combustion-related sources. Consistent with findings from studies of smokers and individuals exposed to diesel engine exhaust, reduced levels of cysteine-derived HSA-Cys^34^ adducts in this cohort reflect alterations in systemic thiol redox balance under conditions of sustained OS [46].

#### 4.2.2. HSA-Cys^34^ Homocysteine (*S*-hCys)

Homocysteine (hCys) is a key intermediate in the metabolism of methionine, an essential amino acid. It is formed through the demethylation of methionine and is involved in two major metabolic pathways: remethylation to regenerate methionine, and transsulfuration to produce cysteine [63]. These pathways are crucial for maintaining the cellular redox balance and supporting vital biochemical processes, including DNA synthesis and methylation.

Abnormally elevated levels of hCys in plasma, a condition termed hyperhomocysteinemia, have been implicated in the pathogenesis of numerous diseases, such as cardiovascular disorders, neurodegenerative conditions, and thrombosis, through mechanisms involving OS, endothelial dysfunction, and inflammation [64].

Grigoriyan et al. observed lower levels of Cys^34^ disulfides of hCys, namely *S*-hCys+C (H_3_ () /*z* 860.77) and *S*-hCys-H (−O ()*m*/*z* 850.10), in colorectal cancer cases [47]. These were either methylated (860.77) or dehydrated (850.10) at an additional site on the T3 peptide. hCys is also a central intermediate in one-carbon metabolism. The remethylation of hCys to methionine and subsequently to *S*-adenosylmethionine, a key donor for DNA methylation, is linked to various cancers, including colorectal cancer. While meta-analyses have generally linked elevated hCys levels with increased colorectal cancer risk, discrepancies have been reported that are speculated to be due to age and dietary differences, as prior studies included mostly older populations (mean age ~61 years) [47].

Occupational studies further revealed that with exposure to diesel engine exhaust, *S*-hCys (*m*/*z* = 856.10) and its methylated derivative *S*-hCys+C (H_3_ ()*m*/*z* = 860.77) were significantly lower [45], while in benzene-exposed workers, higher levels of Cys^34^ disulfides of hCys, namely homocysteine (hCys, 850.10 with loss of H_2_O and 856.43 with NH_2_→OH), were observed, suggesting pollutant-specific redox alterations [39].

Consistent with these patterns, *S*-hCys adducts were significantly lower in lung cancer cases than in controls, indicating disruption of homocysteine metabolism and thiol–disulfide homeostasis. This reduction, together with increased Cys^34^ direct oxidation products, suggests impaired one-carbon and transsulfuration pathways that weaken antioxidant capacity and contribute to the OS associated with PM_2.5_ and PAH exposure [46].

#### 4.2.3. HSA-Cys^34^ Glutathione (*S*-GSH)

Glutathione (GSH) is an LMW thiol tripeptide composed of glutamate, cysteine, and glycine and represents the most abundant non-protein thiol in all mammalian tissues, providing a primary defense against OS [65]. Beyond direct ROS scavenging capacity, GSH plays a pivotal role in maintaining redox homeostasis through its reversible oxidation-reduction cycle (GSH/GSSG) [61,66]. Fluctuations in this redox couple provide a biochemical mechanism linking ROS generation to reversible modifications of protein thiols [61]. One such important modification is protein *S*-glutathionylation (*S*-GSH), characterized by the formation of a mixed disulfide bond between GSH and protein cysteine residues [67].

GSH forms reversible mixed disulfides with HSA-Cys^34^ via non-enzymatic thiol-disulfide exchange, contributing to extracellular redox buffering that limits irreversible HSA-Cys^34^ oxidation [34]. Although extracellular GSH concentrations (2–20 µM) [68] are substantially lower than intracellular levels (0.5–10 mM) [68], circulating GSH functions as an important antioxidant sink that participates in redox buffering through reversible protein modification, particularly on abundant plasma proteins such as HSA. Importantly, levels of *S*-GSH are a sensitive systemic redox metric of the extracellular redox environment, capturing the dynamic equilibrium between glutathione availability and its depletion under OS.

In previous work, we reported that *S*-GSH levels were positively associated with maternal O_3_ exposure during the third trimester [42], suggesting a compensatory antioxidant response (i.e., upregulation of GSH synthesis), whereas levels were significantly lower in patients with COPD and IHD, which was consistent with GSH depletion observed in the lung epithelium of these patients [40]. This is consistent with previous findings of patients with COPD exhibiting reduced levels of endogenous GSH in bronchoalveolar lavage fluid and reduced levels of glutamylcysteine ligase, a crucial enzyme for glutathione synthesis (the rate-limiting enzyme), in the bronchial epithelium and alveolar macrophages [40].

Lu et al. also reported significantly lower *S*-GSH levels in female, non-smoking, coal and wood stove users compared with electric or gas stove users. These results were interpreted as evidence of depletion of intracellular GSH and its precursor γ-glutamyl cysteine [48]. Interestingly, Wong et al. reported higher levels of *S*-GSH in workers exposed to diesel engine exhaust, together with alterations in other Cys^34^ adducts related to antioxidant activity, indicating that diesel exposure is associated with changes in systemic detoxification- and antioxidant-related processes, rather than thiol depletion alone [45].

#### 4.2.4. HSA-Cys^34^ Cysteinylglycine (*S*-CysGly)

Cysteinylglycine (CysGly), a dipeptide precursor of glutathione, can form mixed disulfide adducts with HSA-Cys^34^ under oxidative conditions [65]. Although less abundant than cysteine, CysGly reacts efficiently with Cys^34^ (rate constant ~0.6 M^−1^ s^−1^) and participates in dynamic redox cycling.

CysGly is produced extracellularly through the enzymatic degradation of GSH by γ-glutamyl transpeptidase (GGT) and dipeptidases, which remove the γ-glutamyl moiety and further split the resulting dipeptide into cysteine and glycine, which are recycled for GSH resynthesis. Therefore, the *S*-CysGly adducts reflect both the oxidative status of the plasma redox system and GSH turnover.

In adducts measured in newborn DBS samples, *S*-CysGly was positively associated with maternal PM_2.5_ exposure in the first trimester [42], while lower *S*-CysGly (-H−O) levels have been reported in lung cancer cases, suggesting a perturbation in glutathione metabolism, possibly due to depletion of intracellular GSH [49]. Interestingly, the ratio of *S*-CysGly to *S*-GSH was higher in all solid fuel-exposed groups than in the controls, with smoky coal showing a significantly stronger effect than wood fuel or smokeless coal [48]. This indicates that γ-glutamyl transpeptidase activity may play a role in the reduction in circulating GSH via catabolism. In contrast, *S*-CysGly levels were significantly higher in workers exposed to diesel exhaust, again highlighting pollutant-specific effects on systemic thiol metabolism [45].

Grigoryan et al. similarly reported lower *S*-CysGly levels observed in non-Hodgkin lymphoma cases, particularly in males, suggesting disrupted extracellular GSH recycling and OS-induced thiol depletion [43]. Collectively, *S*-CysGly adducts serve as sensitive indicators of glutathione pathway imbalance and systemic redox perturbation across the environment-disease continuum.

#### 4.2.5. HSA-Cys^34^ γ-Glutamylcysteine (*S*-γ-GluCys)

γ-Glutamylcysteine (γ-Glu-Cys) is a dipeptide intermediate in GSH biosynthesis. It is synthesized from glutamate and cysteine by γ-glutamylcysteine synthetase, which is the rate-limiting enzyme in the GSH synthesis pathway [69]. With its free thiol group, γ-Glu-Cys can directly engage in redox buffering and thiol-disulfide exchange reactions, thus maintaining antioxidant defenses when GSH levels are depleted. Disruptions in γ-Glu-Cys metabolism can impair GSH biosynthesis and redox homeostasis, therefore implicated in OS-related diseases, including neurodegeneration, cardiovascular disease, and cancer [70,71,72,73].

We previously reported that *S*-γ-GluCys was positively associated with PM_2.5_ but negatively associated with O_3_ exposure [42]. Since γ-GluCys participates in maintaining antioxidant defense, depletion due to O_3_ exposure indicates OS-mediated depletion of the plasma thiol pool. In contrast, elevation with PM_2.5_ may be due to compensatory upregulation of the glutathione pathway. Similarly, Lu et al. found significantly lower *S*-γ-GluCys levels in women using coal or wood fuels compared with electric and gas users, consistent with glutathione pathway depletion under chronic air pollution exposure [48].

Consistent with these findings, Grigoryan et al. reported that *S*-γ-GluCys was significantly less abundant in non-Hodgkin lymphoma cases than in controls, especially in males, indicating depletion of GSH precursors and impaired antioxidant defense under prolonged OS [43]. The *S*-γ-GluCys adduct was associated with *S*-CysGly and *S*-GSH in cluster analyses, suggesting coordinated dysregulation of the extracellular GSH turnover pathway mediated by γ-glutamyl transpeptidase. These collective observations show that *S*-γ-GluCys can serve as a sensitive plasma biomarker of OS, redox imbalance, and impaired thiol homeostasis associated with environmental exposures and disease processes.

#### 4.2.6. HSA-Cys^34^ Methanethiol (*S*-Methanethiol)

Methanethiol is a microbial co-metabolite derived from methionine catabolism or hydrogen sulfide methylation by gut microbiota. It forms a disulfide with oxidized Cys^34^ (Cys^34^-SOH) via condensation and water loss [74].

Grigoryan et al. observed elevated *S*-methanethiol (*m*/*z* 827.09) in colorectal cancer [47], aligning with increased fecal methanethiol levels reported in colorectal cancer patients [75] and in male non-Hodgkin lymphoma cases [43]. These findings suggest that enhanced microbial sulfur metabolism and redox imbalance are associated with tumor-linked dysbiosis.

In contrast, we previously observed that the *S*-methanethiol adduct is negatively associated with NO_2_ exposure during the first trimester of pregnancy. This finding indicates that maternal air pollution exposure during early pregnancy is associated with altered biology that is associated with neonatal small-thiol adduct profiles measured at birth [42].

#### 4.2.7. HSA-Cys^34^ N-Acetyl Cysteine (*S*-NAC)

NAC is a precursor of cysteine and GSH that is integral in antioxidant defense. *S*-NAC was significantly lower in prospective lung cancer cases than in controls [49], with decreased *S*-NAC adduct levels indicative of diminished redox capacity, particularly with smoking-induced OS.

Further studies with DNA methylation data have linked low *S*-NAC with hypomethylation at smoking-related CpG sites, linking oxidative imbalance to epigenetic changes. These findings provide the first in vivo evidence that decreased *S*-NAC adducts may serve as early plasma biomarkers of OS and lung cancer risk [49].

Importantly, small thiol adducts of HSA-Cys^34^ are reversible and represent a dynamic component of plasma redox homeostasis. These thiol-disulfide exchange reactions with LMW thiols can regenerate the free thiol form of HSA-Cys^34^ [76,77]. In humans, oral N-acetylcysteine (NAC) supplementation has been shown to facilitate this process by restoring mercaptoalbumin and enhancing plasma antioxidant capacity [76,77]. Beyond NAC, cysteine supplementation has also been shown to regenerate mercaptoalbumin levels but is less effective in maintaining Cys^34^ in free form; its oxidation product, cystine, rapidly reacts back with Cys^34^. More recently, NAC amide (AD4/NACA) and thioredoxin mimetic (TXM) peptides have been shown to effectively regenerate the free thiol form of HSA-Cys^34^ and enhance its antioxidant activity [78]. These findings highlight the potential of targeting Cys^34^ redox cycling as a precision medicine strategy to restore albumin antioxidant function under conditions of oxidative distress [76,77].

### 4.3. Reactive Electrophiles

#### 4.3.1. HSA-Cys^34^ Crotonaldehyde (*S*-Crotonaldehyde)

Crotonaldehyde is a reactive α,β-unsaturated aldehyde produced by oxidation of membrane lipids [79]. As a reactive carbonyl species, *S*-crotonaldehyde serves as both a marker of and a mediator of oxidative damage. *S*-crotonaldehyde was reported to be significantly elevated in workers exposed to benzene (a strong promoter of ROS) [39] and in colorectal cancer cases compared to controls, supporting its role as a biomarker of lipid peroxidation and electrophilic stress [47].

Mechanistically, crotonaldehyde is associated with redox-inflammatory signaling and carcinogenic processes. Liu et al. highlighted lipid peroxidation as a central event linking COX-2 induction with colorectal cancer progression via two parallel pathways: (1) enhanced prostaglandin synthesis and (2) impaired degradation of β-catenin (CTNNB1) [80]. Interestingly, *S*-crotonaldehyde (*m*/*z* 835.11) clustered closely with the *S*-methanethiol, indicating a shared biological origin or regulatory response. This co-occurrence supports a plausible model in which gut microbiota invasion triggers localized inflammation, resulting in excessive ROS and *S*-methanethiol formation, leading to oxidative damage and disruption of redox signaling pathways that may contribute to disease initiation and progression [81].

We previously reported significant associations between *S*-crotonaldehyde levels measured in newborn DBS samples and multiple air pollutants (PM_2.5_, PM_10_, O_3_, and NO_2_) during pregnancy [42]. Exposure to O_3_ during the third trimester and the last 30 days of pregnancy was positively associated with *S*-crotonaldehyde, consistent with enhanced lipid peroxidation in the developing fetus under increased oxidative burden late in gestation. In contrast, exposure to PM_2.5_, PM_10_, and O_3_ during the early stages of pregnancy (first and second trimesters) was negatively associated with *S*-crotonaldehyde, suggesting adaptive antioxidant responses in the fetus following earlier oxidative challenges.

Grigoryan et al. reported that *S*-crotonaldehyde was significantly lower in female non-Hodgkin lymphoma cases than in controls, suggesting sex-specific differences in redox responses and disruptions in OS pathways [43]. Together, these findings indicate that *S*-crotonaldehyde reflects a cumulative OS and lipid peroxidation response that together capture a combination of responses to environmental exposures and endogenous redox imbalance in non-Hodgkin lymphoma pathogenesis.

#### 4.3.2. HSA-Cys^34^ Acrolein (*S*-Acrolein)

Acrolein, a reactive α,β-unsaturated aldehyde, is linked to cardiovascular disease risk [82]. Exposures to acrolein can occur through lipid peroxidation (i.e., endogenous production of acrolein) and through exogenous exposures to combustion processes, cigarette smoke, and e-cigarette vapors [83,84]. *S*-Acrolein is readily formed through Michael addition reactions of acrolein with the nucleophilic Cys^34^ thiol. Yano et al. also detected lower levels of *S*-acrolein in newborns of mothers who smoked [50].

## 5. Conclusions and Future Perspectives

HSA-Cys^34^ adductomics provides unique advantages as a biomarker platform for assessing systemic oxidative and electrophilic stress. HSA-Cys^34^ is a sentinel nucleophile in the plasma redox environment, marked by its exceptional reactivity with circulating electrophiles and other small molecules, wide abundance in plasma, and long biological half-life in the blood. The vast diversity of adducts formed at HSA-Cys^34^ captures both a wide range of complex metabolic processes and oxidative responses to exogenous environmental exposures. Site-specific covalent modifications, ranging from reversible thiol-disulfide exchange with low molecular weight thiols to irreversible oxidation reactions and conjugations with reactive electrophiles, capture a more complete and systemic integration of OS and redox imbalance than conventional biomarkers of OS that are central to the pathophysiology of numerous chronic diseases, such as cancer, cardiovascular disorders, and pulmonary conditions. Recent findings, summarized in this review, provide the foundation for applying HSA-Cys^34^ adductomics as a novel biomarker approach for simultaneously quantifying multiple layers of OS and redox biology in single experiments, which is otherwise not possible using traditional biomarker approaches. The complex redox behavior of Cys^34^, such as the formation of sulfinic and sulfonic acids through direct oxidation reactions, varies across exposure sources and disease states, reflecting the nuanced and complex interplay between OS, oxidative distress, and physiological adaptation and regulation captured in the HSA-Cys^34^ adductome. Studies have also revealed pollutant- and disease-specific patterns in thiol-disulfide homeostasis, with changes in mixed disulfides, including cysteine, homocysteine, glutathione, and other small thiols, serving as sensitive indicators of systemic perturbations in redox balance.

As with other omics technologies, there are several challenges using HSA-Cys^34^ adductomics in public health research, including the interpretation of the complex biology captured within adductomics datasets, the difficulty of distinguishing causal relationships from correlative associations, and the need for standardized analytical workflows and curated reference databases for adduct identification. Moving forward, establishing standardized analytical workflows and developing curated adduct libraries with improved annotation strategies will be essential for advancing accurate identification and biological interpretation of the HSA-Cys^34^ adductomics data. In addition, application of machine learning and AI approaches for pattern recognition and biomarker discovery in studies utilizing large, heterogeneous cohorts can enable the identification of exposure-specific signatures and prioritize biologically relevant adduct targets associated with disease risk and progression. These approaches may further enable individualized exposure profiling and improved risk stratification, supporting the development of precision public health strategies.

The integration of adductomics data with other omics platforms, such as metabolomics, lipidomics, proteomics, and genomics, will further enhance the understanding of the molecular networks influenced by OS and provide deeper insight into redox-linked disease mechanisms. HSA-Cys^34^ adductomics represents a promising new omics platform for interpreting redox biology that will provide unprecedented new opportunities in exposomics research.

Together, these advances highlight the potential of HSA-Cys^34^ adductomics as a novel biomarker approach for comprehensively interrogating system-wide OS and perturbations in redox biology within the exposure-disease continuum. With continued advancements in analytical capabilities and integration with other omics biomarker datasets, HSA-Cys^34^ adductomics is well positioned to strengthen OS biomarker research and deepen our understanding of how systemic redox imbalance influences human health and disease.

## Figures and Tables

**Table 1:** Summary of HSA-Cys^34^ adductomics findings across epidemiological and occupational studies showing associations with environmental exposures, lifestyle factors, or disease states.

Adduct	Population	Metric	Reported Value	Adduct Altered	Key Association or Finding	Ref.
*S*-sulfenic acid (*monooxidation*)	benzene-exposed factory workers	PAR	Control: 2.46 Exposed: 2.85	*S*-sulfenic acid ↑	Increase in benzene-exposed workers	[39]
	COPD and IHD	PAR	Controls: 1.21 COPD: 1.04 IHD: 1.37	HSA-Cys^34^-Gln cross-link ↓	Decrease in COPD and IHD	[40]
*S*-sulfinic acid (*dioxidation*)	Smokers vs. non-smokers	PAR	2.25 *	*S*-sulfinic acid ↓ *S*-sulfinic acid +(Na )↓	Decrease in smokers; Negative association with smoking exposure	[41]
	Benzene-exposed factory workers	PAR	Control: 2.41 Exposed: 3.32	*S*-sulfinic acid ↑	Increase in benzene-exposed workers	[39]
	COPD and IHD	PAR	Controls: 1.54 COPD: 1.30 IHD: 1.29	*S*-sulfinic acid ↓	Decrease in COPD and IHD	[40]
			Controls: 0.06 COPD: 0.05 IHD: 0.05	*S*-sulfinic acid +( mCH_3_)↓		
	Prenatal air pollution exposures	PAR ^a^	5.48 *	*S*-sulfinic acid ↑↓	Positively associated O_3_ exposure during 1st trimester, PM_10_ during 2nd trimester and NO_2_ during 3rd trimester Negatively associated with O_3_ during the 3rd trimester and the last 30 days of pregnancy	[42]
	Non-Hodgkin lymphoma	FC	0.83	*S*-sulfinic acid ↓	Decrease in non-Hodgkin lymphoma cases (males)	[43]
	Infants (BPD-PH vs. BPD without PH)	PAR	1.073 *	*S*-sulfinic acid (+CH_3_, −H_2_O) ↓	Decrease in BPD-PH infants compared to infants with BPD without PH	[44]
*S*-sulfonic acid (*trioxidation*)	Benzene-exposed factory workers	PAR	Control: 0.5 Exposed: 1.7	*S*-sulfonic acid ↑	Increase in benzene-exposed workers	[39]
	COPD and IHD	PAR	Controls: 0.22 COPD: 0.214 IHD: 0.184	*S*-sulfonic acid ↓	Decrease in IHD	[40]
	Air pollution exposure in Central London	PAR	Controls: 0.22 COPD: 0.214 IHD: 0.184	*S*-sulfonic acid ↓	Negative association with O_3_ and NO_2_ exposure	[40]
*S*-Cys	Smokers vs. non-smokers	PAR	557.6 *	*S*-Cys ↓	Decrease in smokers; negative association with smoking exposure	[41]
			1.59	*S*-Cys(+Na )↓		
			0.45	*S*-Cys (+K) ↓		
	Diesel-engine factory workers (occupational PM_2.5_)	PAR, FC	0.44 *, 0.91 ^b^	*S*-Cys (NH⟶OH) ↓	Decrease in diesel engine exhaust exposure in factory workers	[45]
	Shanghai Women’s Health Study (Lung cancer in never smokers with air pollution)	PAR, FC	0.0005 *, 0.93	*S*-Cys (−_2_O) ↓	Decrease in lung cancer cases	[46]
			0.002 *,1.03	*S*-Cys (+Na) ↓		
*S*-hCys	Colorectal cancer cohort	PAR, FC	0.73 *, 0.83	*S*-hCys (+CH_3_) ↓	Decrease in colorectal cancer cases	[47]
			0.13 *, 0.82	*S*-hCys (−_2_O) ↓		
	Diesel-engine factory workers (occupational PM_2.5_)	PAR, FC	5.85 *, 0.92 ^b^	*S*-hCys ↓	Decrease in diesel engine exhaust exposure in factory workers	[45]
			0.21 *, 0.77 ^b^	*S*-hCys (+CH_3_) ↓		
	Benzene-exposed factory workers	PAR	Control: 0.10 Exposed: 0.17	*S*-hCys (−_2_O) ↑	Increase in benzene-exposed workers	[39]
			Control: 0.05 Exposed: 0.09	*S*-hCys (NH_2_→OH) ↑		
	Shanghai Women’s Health Study (Lung cancer in non-smokers with air pollution)	PAR, FC	0.2 *, 0.94	*S*-hCys ↓	Decrease in lung cancer cases	[46]
*S*-GSH	Prenatal air pollution exposures	PAR ^a^	15.65 *	*S*-GSH ↑	Positively associated with O_3_ exposure during the 3rd trimester	[42]
	Rural Chinese women using solid fuel	PAR ^a^	Control: 4.59 Smoky: 2.59 Wood: 3.13 Smokeless: 2.88	*S*-GSH ↓	Decrease in coal and wood users compared with gas users	[48]
	COPD and IHD	PAR	Controls: 2.92 COPD: 2.27 IHD: 2.36	*S*-GSH ↓	Decrease in COPD and IHD	[40]
	Diesel-engine factory workers (occupational PM_2.5_)	PAR, FC	0.28 *, 1.22 ^b^	*S*-GSH ↑	DInrease in diesel engine exhaust exposure in factory workers	[45]
*S*-CysGly	Prenatal air pollution exposures	PAR ^a^	0.31 *	*S*-CysGly ↑	Positively associated with PM_2.5_ exposure during 1st trimester	[42]
	EPIC-Italy nested case–control (lung cancer risk)	-	-	*S*-CysGly (−_2_O) ↓	Decrease in lung cancer cases	[49]
	Non-Hodgkin lymphoma	FC	0.94	*S*-CysGly ↓	Decrease in non-Hodgkin lymphoma cases (males)	[43]
*S*-γ-GluCys	Prenatal air pollution exposures	PAR ^a^	0.18 *	*S*-γ-GluCys ↑ ↓	Positive association with PM_2.5_ exposure Negative association with O_3_ exposure	[42]
	Rural Chinese women using solid fuel	PAR ^a^	Control: 2.89 Smoky: 2.02 Wood: 2.45 Smokeless: 1.91	*S*-γ-GluCys ↓	Decrease in coal and wood users compared with gas users	[48]
	Non-Hodgkin lymphoma	FC	0.71	*S*-γ-GluCys ↓	Decrease in non-Hodgkin lymphoma cases, particularly in males	[43]
*S*-Methanethiol	Colorectal cancer cohort	PAR, FC	0.12 *, 1.20	*S*-Methanethiol ↑	Increase in colorectal cancer cases	[47]
	Non-Hodgkin lymphoma	FC	1.39	*S*-Methanethiol ↑	Increase in non-Hodgkin lymphoma cases (males)	[43]
	Prenatal air pollution exposures	PAR ^a^	0.27 *	*S*-Methanethiol ↓	Negative association with NO_2_ exposure during 1st trimester	[42]
*S*-NAC	EPIC-Italy nested case–control (lung cancer risk)	-	-	*S*-NAC ↓	Decrease in lung cancer cases	[49]
*S*-Crotonaldehyde	Benzene-exposed factory workers	PAR	Control: 0.28 Exposed: 0.57	*S*-Crotonaldehyde ↑	Increase in benzene-exposed workers	[39]
	Colorectal cancer cohort	PAR, FC	0.32 *, 1.13	*S*-Crotonaldehyde ↑	Increase in colorectal cancer cases	[47]
	Prenatal air pollution exposures	PAR ^a^	1.38 *	*S*-Crotonaldehyde ↑↑	Positive association with O_3_ exposure (3rd trimester and last 30 days of pregnancy) egative association with PM_2.5_, PM_10_, and O_3_ during early stages of pregnancy (first and second trimesters)	[42]
	Non-Hodgkin lymphoma	FC	0.79	*S*-Crotonaldehyde ↓	Decrease in non-Hodgkin lymphoma cases, particularly in females	[43]
*S*-Acrolein	Smoking mothers vs. non-smoking mothers	FC	0.82 ^b^	*S*-Acrolein ↓	Decrease in newborns with smoking mothers	[50]

PAR, peak area ratio = [(adduct peak area/HK peptide peak area) × 1000]; values are reported as mean PAR unless otherwise specified; ^a^ median PAR; FC, fold change (case/control); ^b^ fold change (exposed/unexposed); * PAR of the entire study population.; Direction of change: ↑ indicates a positive association, and ↓ indicates a negative association.

## Data Availability

No new data were created or analyzed in this study. Data sharing is not applicable to this article.

## Abbreviations

The following abbreviations are used in this manuscript:

HSA	Human serum albumin
OS	Oxidative stress
ROS	Reactive oxygen species
RNS	Reactive nitrogen species
LPO	Lipid peroxidation
TAC	Total antioxidant capacity
SOD	Superoxide dismutase
MDA	Malondialdehyde
4-HNE	4-hydroxynonenal
GSH	Glutathione
GSSG	Glutathione dilsulfide
GST	glutathione S-transferase
RCS	Reactive carbonyl species
LMW	Low molecular weight
COPD	Chronic obstructive pulmonary disease
IHD	Ischemic heart disease
DBSs	Dried blood spots
BPD-PH	Bronchopulmonary dysplasia-associated pulmonary hypertension
CSO	Cumulative supplemental oxygen
